# Examining the accuracy of trackways for predicting gait selection and speed of locomotion

**DOI:** 10.1186/s12983-020-00363-z

**Published:** 2020-05-27

**Authors:** Andres Marmol-Guijarro, Robert Nudds, Lars Folkow, Jonathan Codd

**Affiliations:** 1 Faculty of Biology, Medicine & Health, University of Manchester, Manchester, UK; 2 Department of Arctic and Marine Biology, University of Tromsø - The Arctic University of Norway, Tromsø, Norway

**Keywords:** Gait, Locomotion, Arctic, Biomechanics, Snow, Track, Speed

## Abstract

**Background:**

Using Froude numbers (*Fr*) and relative stride length (stride length: hip height), trackways have been widely used to determine the speed and gait of an animal. This approach, however, is limited by the ability to estimate hip height accurately and by the lack of information related to the substrate properties when the tracks were made, in particular for extinct fauna. By studying the Svalbard ptarmigan moving on snow, we assessed the accuracy of trackway predictions from a species-specific model and two additional *Fr* based models by ground truthing data extracted from videos as the tracks were being made.

**Results:**

The species-specific model accounted for more than 60% of the variability in speed for walking and aerial running, but only accounted for 19% when grounded running, likely due to its stabilizing role while moving faster over a changing substrate. The error in speed estimated was 0–35% for all gaits when using the species-specific model, whereas *Fr* based estimates produced errors up to 55%. The highest errors were associated with the walking gait. The transition between pendular to bouncing gaits fell close to the estimates using relative stride length described for other extant vertebrates. Conversely, the transition from grounded to aerial running appears to be species specific and highly dependent on posture and substrate.

**Conclusion:**

Altogether, this study highlights that using trackways to derive predictions on the locomotor speed and gait, using stride length as the only predictor, are problematic as accurate predictions require information from the animal in question.

## Background

Understanding what speeds of locomotion animals choose during interactions with conspecifics (i.e. social or reproductive behaviour), other species (i.e. predation or predator avoidance), and when moving through an often-changing environment is paramount to better understanding their biology. Studies of terrestrial animal locomotion, however, are overwhelmingly conducted under laboratory conditions using treadmills [1]. Treadmill studies have facilitated great insight into the biomechanics of locomotion and using this approach the correlation between kinematic parameters like stride length (*l*_stride_), stride frequency (*f*_stride_) stance (*t*_stance_) and swing (*t*_swing_) time with speed (*U*) has been widely reported in the literature across a range of species. For example; treadmill kinematic data exists on a wide range of mammals including polar bears [2]; horses [3]; otters [4]; deer [5] cats [6], rodents [7, 8] and monkeys [9]. However, perhaps the most comprehensive research into animal locomotion has been conducted in birds [10–17]. The focus on avian biomechanics is likely due to birds evolutionary link to their theropod ancestors, being bipedal, easy to train, experimentally tractable and exibiting a wide range of adaptations. For example, the Svalbard rock ptarmigan (*Lagopus muta hyperborea*) has been extensively studied for locomotor adaptations related to energy savings upon gait change [18], sexual selection [19], efficient load carriage [20] and ontogeny [21].

Studying the locomotion of wild animals in their natural enviroment can be challenging, many animals are elusive and fieldwork can be protracted, expensive and prone to a wide range of factors that cannot be controlled. Trackways are one way to circumvent these issues and may provide insight into the biology of animals in the absence of the animal themselves. To this end, tracks have been used to help understand aspects of extinct fauna such as their diversity, the description of new ichnotaxa, and to gain inference into morphological, behavioural, and ecological aspects of the trackmakers (e.g. [22–26]). Trackways have also provided evidence of key evolutionary events such as the transition of tetrapods from water to land ( [27], see [28]) and the first bipedal hominids (e.g. [29–31]). Outside of the evolutionary insights, perhaps the most common usage of the information gleaned from trackways relates to gait selection and speed (e.g. [23, 32–35]). An established concept for extracting speed (and gait) from trackways uses the *Fr* [10, 36], defined as:

1 $$ Fr=\frac{U^2}{gh} $$

Where *U* is speed, *g* is the acceleration due to gravity and *h* is the functional hip height. *Fr* is a dimensionless number that by equalising the centripetal to gravitational force ratio allows the locomotion of terrestrial animals to be compared equally across all sizes. Geometrically similar animals of different sizes will move in a dynamically similar way at any given *Fr*. In practice, not all animals are geometrically similar, but it was argued that despite this, the ratio of stride length (*l*_stride_) to *h* gave a highly predictable relationship across a broad size range of mammals and birds [10, 11]. By using this empirically derived relationship with the *Fr* concept, it was further suggested [10] that the forward *U* of a terrestrial animal can be calculated from:

2 $$ U=0.25{g}^{0.5}{l}_{\mathrm{stride}}^{\kern3.5em 1.67}{h}^{-1.17} $$

*Fr* may also allow the *U* at which gait transition occurs (e.g. walking to running) to be estimated [36]. Alexander [10] and Thulborn [37] suggest that gaits will shift from walking to a bouncing gait (e.g. trotting) when *l*_stride_/*h* reaches 2.0, and the transition from trotting to running (or galloping) at an *l*_stride_/*h* of 2.9. Eq. 2 is therefore probably applicable to walking animals only [38]. For highspeed gaits where *l*_stride_/*h* is greater than 2.9 the following is advocated as being more appropriate for estimating *U* [23, 38]:

3 $$ U={\left[ gh\ {\left(\frac{l\mathrm{stride}}{1.8\ h}\right)}^{2.56}\right]}^{0.5} $$

For trotting *U* is better estimated by the mean of predictions derived from eqs. 2 & 3 [23]. Irrespective of the equation used, the reliability of estimates of *U* may be compromised if there is lack of certainty on *h* –in particular in extinct animals where *h* is not available [39–42]– and the use of *l*_stride_ boundaries that may not be compatible with bipedal gaits [39]. Trackways are therefore restricted in the information that they can provide as much of the information needed for accurate locomotion analysis, such as leg morphology and stride frequency, depends on data from the animal itself. It is worth remembering that anecdotally the vast majority of extant animal movement does not leave evidential tracks. Aside from seeing occasional footprints in the sand or on muddy ground, overwhelmingly animals are not moving over substrates where their feet will leave lasting impressions. An exception to this is locomotion over snow which will, in the vast majority of cases, leave tracks. Regions of the world, like the Arctic are seasonally covered in snow which provides an opportunity to examine trackways and the kinematics of locomotion in context of the real-world influence of variations in substrate. Svalbard rock ptarmigan are endemic to the high Arctic Archipelago of Svalbard meaning they spend approximately half a year locomoting over snow and they are also one of the few species in which a comprehensive laboratory treadmill dataset exists which can be used for comparison. Recently one of the first comparisons of the kinematics of locomotion under field and laboratory treadmill conditions was undertaken in the Svalbard rock ptarmigan [1]. The kinematics of locomotion were conserved for ptarmigan moving in the field and during laboratory treadmills studies but only for walking and aerial running gaits. Important differences were found when the birds were grounded running, with the birds taking faster and shorter steps in the field when compared to the movement on the treadmill. These kinematic differences were attributed to differing substrate when moving over snow compared to a treadmill belt [1]. Our ptarmigan studies also highlighted the importance of understanding the influence substrate can have on locomotion [1]. Studies in extant animals have demonstrated that substrate can influence the neuromuscular control of locomotion to maintain stability [43–45] and can affect the energetic cost [46, 47] and the speed [48] of locomotion. Furthermore, despite the obvious links between trackways and the ground, substrate is rarely considered when inferences into speed and gait are made from tracks. Not taking any potential effect of substrates into account is surprising as information derived from tracks depends more on the substrate properties than other potentially important variables like the anatomy of the foot itself [49]. Substrate effects can be difficult to assess under some situations. For example, the water content of the substrate at the time of trackway formation is uncertain. However, when substrate is considered it is most often examined in terms of the formation of the physical tracks themselves (see [49]). Consideration of substrates and tracks has also been used to demonstrate that in extant species foot morphology can vary with stance and gait [50, 51] and highlighted the interaction of the feet with different sedimentary substrates [52, 53].

The principle objective of our study was to develop a species-specific model to examine gait and speed predictions directly from *l*_stride_ of trackways of the Svalbard rock ptarmigan. The accuracy of these trackway derived speed predictions and gait transitions was determined by ground truthing data extracted from videos of the birds taken as the tracks were being made. Finally, a comparison between the predictions obtained using our ptarmigan species-specific model and existing *Fr* based models [10, 23, 38] were made to elucidate the accuracy of each approach. Further, we discuss how reliable information extracted from trackways is for examining the predicted speed of locomotion in both extant and extinct animals.

## Results

**Fig. 1:**
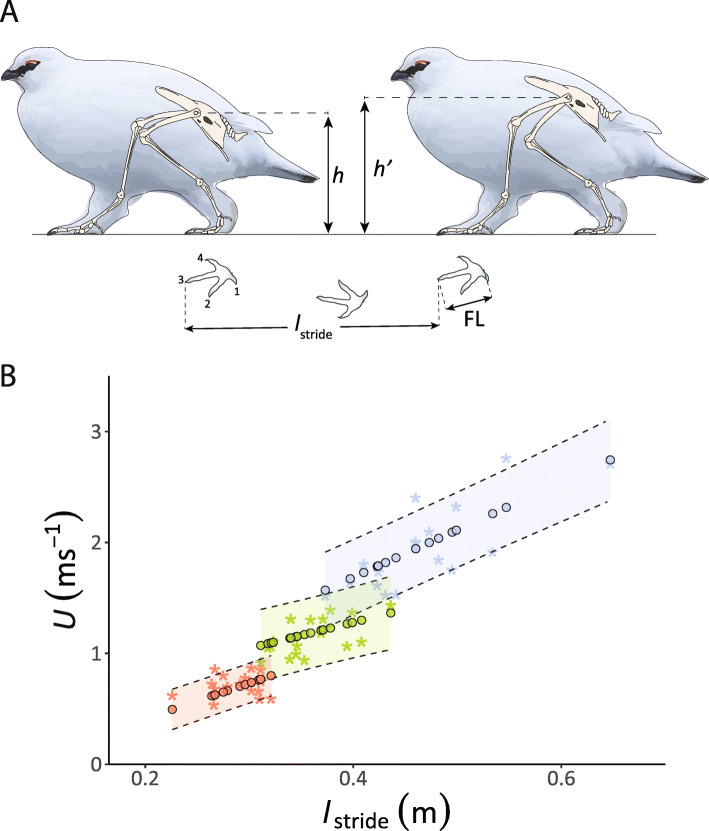
Trackways of the Svalbard Rock Ptarmigan. **a** length (FL), Stride length (*l*_stride_) and Hip Height (*h*) are used to estimate speed. *h* is the distance to the top of the hip perpendicular to ground. This measurement however, is entirely dependent on accurate joint angles of the leg bones. As illustrated, a small alteration in joint angle results in different hip height measurements (*h* < *h*’). It has been suggested that four times FL can be used as a proxy of effective hip height however this method is also prone to error when applied to extinct animals where there is no information on the joint angles. Digits are numbered 1–4 for analysis as indicated. **b** Speed (*U)* predictions from stride length (*l*_stride_) using the ptarmigan species-specific model. The stars represent the data determined from video recordings and the filled circles represent the predicted value for a given *l*_stride_ for 50 birds calculated from trackways that corresponded to the video recordings. Red, green and blue represent walking, grounded running and aerial running gaits, respectively. The coloured area delimited by the dashed lines represents the predictive interval for the lines of best fit (corresponding to the filled circles) described by the following equations: *U* = 3.20 *l*_stride_ – 0.23 (walking), *U* = 2.34 *l*_stride_ + 0.34 (grounded running) and for aerial running is *U* = 4.29 *l*_stride_ – 0.03 (aerial running)

As expected, *U* increased linearly with increasing *l*_stride_ across all gaits (Fig. 1a, b), although the amount of variation in *U* explained by *l*_stride_ during grounded running was much lower than that for the other two gaits (walking: *F*_1, 46_ = 86.25, *r*^2^ = 0.64, *p* < 0.001; grounded running: *F*_1, 54_ = 14, *r*^2^ = 0.19, *p* < 0.001; aerial running: *F*_1, 59_ = 133.2, *r*^2^ = 0.69, *p* < 0.001). For walking trackways, the corresponding regression model predicts ptarmigan walking speeds in the range 0.49 ± 0.18 to 0.80 ± 0.18 ms^− 1^. For the grounded running trackways, the model predicts birds using speeds that range from 1.07 ± 0.33 to 1.36 ± 0.33 ms^− 1^. The predictions of *U* for aerial running suggest ptarmigan use this gait in a speed range from 1.57 ± 0.34 to 2.74 ± 0.37 ms^− 1^ (Fig. 1).

**Fig. 2:**
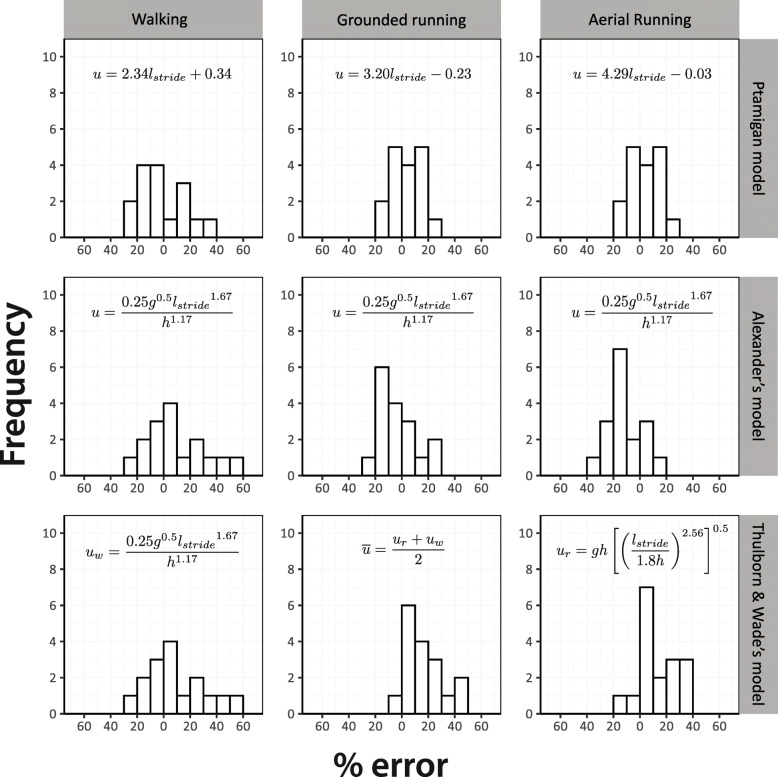
Error estimation for the ptarmigan species-specific regression models and the Alexander (1976), and Thulborn, and Wade (1984) *Fr* derived equations. Bars to the left of zero represent estimations of *U* below the measured *U*, and vice versa. Note that the error estimates from Alexander (1976) and Thulborn, and Wade (1984) for walking gaits are identical because they use the same equation for this gait. Errors have been binned to 10

Predicted *U* for each of the 50 birds in this study, were more accurate using our model than those of Alexander [10] and Thulborn and Wade [23]. The error (Eq. 4) associated with predictions derived from the ptarmigan specific model for the 50 birds within all gaits were between 0 and 30% (mean error = 11.8, SD = ± 8.2), except one that was 35% (Fig. 2). In contrast, the errors related to the predicted *U* from Alexander [10] and Thulborn and Wade [23] were 0 to 55% (Alexander: mean error = 14.4, SD = ± 11.0; Thulborn & Wade: mean error = 17.0, SD = ± 13.8), in both cases (Fig. 2). Within the three gaits, walking was associated to the largest errors, although they had less variation.

## Discussion

The ptarmigan specific model for the walking and aerial running gait accounted for a moderate amount of the variation in *U* (64 and 69%, respectively), and for the grounded running gait was lower, accounting for less that 20% of the variation. The inability to predict *U* for the grounded running gait with any confidence is likely due to the influence of substrate, which particularly effects this intermediate gait [1]. All terrestrial locomotion involves interaction with a substrate. The slow walking speeds, however, are thought to negate the influence of substrate on locomotion kinematics as they provide greater resistance to external ground perturbations acting on the centre of mass [54, 55] whilst fast running speeds where only possibly over hard substrates anyway [1]. The reason there is much more variability in *U* during the grounded running gait which reduces the ability to make accurate predictions is that a grounded running gait is used as a mechanism to move faster while also maintaining stability, by cancelling the effects of uneven ground and a changing substrate through increased times of contact of the foot to the ground and a more compliant leg [11, 45, 56]. For ptarmigan moving over snow, modifications in *l*_stride_ during grounded running are required to prevent falls [1] since shorter steps improve locomotion over slippery surfaces by keeping the centre of mass above the supporting limbs [57, 58].

The ptarmigan specific model represents the best-case scenario in terms of using *l*_stride_ to predict speed in that we were able to match these data to accurate morphological measurements of hip height and also to double check predictions against real speeds calculated from simultaneous video recordings. Despite these advantages the ability to predict speeds still lacks of accuracy, likely accounted for by the inherent variation in kinematic parameters within a gait (as demonstrated in Fig. 1a). After comparing the error estimates between the predicted *U* of the three models (Alexander [10] and Thulborn and Wade [23] and the current study) against the measured *U* from the 50 video-recordings, the error associated with the predictions were lower using our model. This result is not surprising, as our models were built upon previously measured and validated data for the three gaits specifically from Svalbard ptarmigan. Therefore, it deals only with the uncertainty associated with the intraspecific variation within the Svalbard ptarmigan. On the other hand, the equations from Alexander [10] and Thulborn and Wade [23] were derived from kinematic data of several extant taxa, most of them quadrupedal mammals, that possess diverse geometries and move in a subtly different way. Despite the relatively low error for the ptarmigan speed estimates using the three models (up to 55% in our study compared to up to 200% reported elsewhere [59–61]) they would still lead to inaccurate predictions on the metabolic cost of locomotion, in particular at walking speed where the predictions would change drastically [18].

Identifying gaits in the absence of the animal solely from footprints is challenging if no other information is available. However, approximations of the relative *l*_stride_ and the dimensionless *Fr* before a gait change was suggested by Alexander [10] as a means that allowed researchers to infer which gait an animal was using. Alexander [10], in his paper on dinosaur footprints, proposed that the transition from a walking gait to trotting or running occurs approximately at *Fr* of 0.6 and at a relative *l*_stride_ (i.e. *l*_stride_ divided by hip height) of 2.0. His suggestions were based on a comparison of extant mammals under the principle of dynamic similarity. Our results partially agree with those intervals. By estimating relative *l*_stride_ using the mean hip height of 0.1727 m for a male ptarmigan as hip height doesn’t change [19], our data suggest that ptarmigan shift from walking to grounded running at a maximum relative *l*_stride_ taken by a walking bird of 2.03 (*l*_stride_ = 0.35 m) (Fig. 1b). The fastest walking ptarmigan was recorded moving at a *Fr* of 0.5 (*U* = 0.92 ms^− 1^) (Fig. 1b). These data support the widely accepted idea that animals shift from a pendular to a bouncing gait at similar relative speeds when moving in a dynamically similar fashion [11, 36]. Consequently, it is not surprising that our predictions for the walking gaits fall within these proposed boundaries. In a further attempt to distinguish between trotting and running gaits, Thulborn and Wade [23] extended the scope of the Alexander [10] method by incorporating the transition from trotting to running at a relative *l*_stride_ of 2.9 [23]. However, this conclusion was based on records of ungulates shifting from trotting to galloping/running [39] and therefore they may not be applicable to the bipedal gaits of birds. Indeed, differences in the leg kinematics of birds and mammals moving at similar *Fr* were shown by Lees et al. [62]. Our results suggest that ptarmigan shift from grounded to aerial running at a lower relative *l*_stride_ ranging from 1.93 to 2.57. Within the existing literature, however, there are conflicting results even among avian species. For example, a closer look at the relative *l*_stride_ vs. relative velocity plot in Gatesy and Biewener [11] shows similar values for ostriches (relative *l*_stride_ = 2.44) and rhea (relative *l*_stride_ = 2.37) at the point of change to aerial running. Abourachid and Renous [13] found that relative *l*_stride_ at the transition to aerial running is 2.02 and 1.76 for ostriches and emus, respectively. In contrast, turkeys and guinea fowls show a higher relative *l*_stride_ of 3.14 and 3.73 at the transition, respectively [11]. Such differences suggest that posture needs to be considered if a diagnosis of gait is to be made solely on the footprints of extant animals and casts doubt on using this approach for extinct animals.

### Implication for trackways of extinct animals

Obtaining accurate information from trackways in relation to speed and gait choice is difficult even for extant animals for which morphological measurement, and matched field and laboratory treadmill data exist. However, meaningful data are only possible if researchers are able to measure locomotor kinematics and gait selection of the animal concomitant with analysis of the trackways. Therefore, in order to obtain accurate predictions of the biomechanics of locomotion from trackways this requires data from the animal themselves in order to ground truth the data. Unfortunately, the uncertainty about morphology of extinct bipeds lead to several assumptions that may compromise speed estimates derived from hip height when using Alexander [10] and Thulborn and Wade [23] methods, in particular if they are derived from trackways alone. When using trackways, posture is often estimated by deriving hip height as approximately four times foot length (e.g. [10, 23, 32–34, 63, 64]). However, such postural estimates can vary by a factor of 1.5 or more [59, 60], and might be further affected if the trackmakers moved over compliant substrates, such as sedimentary river banks or mud, creating mismatches between the “real” foot morphology of the trackmaker compared to the imprinted track that may be relatively smaller [49, 52]. These methodical limitations are often acknowledged in such studies and were recognized by Alexander himself [60, 61]. Numerous efforts to incorporate biomechanical principles to improve the predictive models have been done in regards to posture on specimens where fossilized skeletons are available ([10, 40–42], reviewed in [61, 65]). researchers have also recognized the sensitivity to assumptions on hindlimb anatomy [41, 65], including assumptions on muscular mass and power [66]. Hence, irrespective of the equation used, the reliability of estimates of speed are compromised by the lack of certainty in the foot length–hip height ratio of an extinct trackmaker [39–42, 59] and the use of stride *l*_stride_ boundaries that may not be compatible with bipedal gaits [39]. Trackways are therefore restricted in the information that they can provide as much of the information needed for accurate locomotion analysis, such as leg morphology and stride frequency, depends on data from the animal itself.

The lack of certainty on the morphology of the track maker raises the conundrum that if data from the animal is required when it is making the tracks to calculate speed and gait choice, why keep trying to get this information from the trackways alone? There is no doubt that tracks offer an unique record of behavioural and evolutionary aspects of extinct fauna, including discrete locomotor events like transition from slow to fast locomotion [34, 35, 67]. The caveat is that the trackways on their own cannot provide a complete and accurate quantification of the animals’ speed and gait [39, 60, 61]. For the ptarmigan 34% of tracks would have been unable to be classified into a given gait and speed based on the trackways *l*_stride_ alone because of the overlap when the birds were transitioning between either walking to grounded running or grounded running to aerial running gaits. It should be noted that we are only able to accurately assess the error in predicting just from tracks for the ptarmigan as it represents a ‘best case scenario’ where we have all possible information. Many birds and other animals use transitional gaits, suggesting this issue is likely widespread in extinct forms as well. Other unknowns, not quantified in the current study, but likely to further cloud the inferences from tracks in isolation are the influence of sex differences on the kinematics of locomotion [16, 68–70] and ontogenetic influences [21] all of which cannot be quantified in extinct animals. Inferences into the biology of extinct forms commonly suffers from large errors [60, 61, 71] and rely on numerous assumptions when extrapolating from extant to extinct forms [65, 72, 73].

## Conclusion

Here we calculated the speed of locomotion of the Svalbard rock ptarmigan measured from video recordings of ptarmigan while moving over snow, and immediately after estimated using *l*_stride_ from trackways left by the birds, using one species specific model that accounted for body size and two more general models based on dynamic similarity of locomotion [10, 23]. After ground truthing the measured speed with estimated speeds with three models giving estimates with 30–35% of error, 55 and 55% error, for the species-specific model (our study), and the models of Alexander [10], and Thulborn and Wade [23], respectively. Similarly, more than one third of the tracks were not able to be assigned to a specific gait due to speed ranges overlapping between gaits. Our results suggest that speed and gait estimates are not reliable when they are only based on trackways. A better understanding of the interaction between tracks and locomotion is likely to be useful for future studies, in conjunction with biologging data on activity budgets, for examining how substrates influence the metabolic cost of locomotion. For example, to improve the predictive power of all models, in particular for transitional gaits, future analysis including a quantitative assessment of the hardness, density and roughness of the substrate immediately after the impression of trackways are made would be beneficial.

## Methods

### Animals and data collection

All data were collected in Adventdalen valley (78°13′18″N, 15°38′30″E) and the surrounding side valleys in the Svalbard Archipelago, during Spring, 22nd April to 4th May 2017. At this time of year, the ground is covered by snow and daylight persists for 24 h per day. Svalbard ptarmigan were already at their summer weight [74]. Male birds were spotted with binoculars, identified by their distinctive calls and secondary sexual characteristics; red supraorbital combs and thick black eye stripes. Birds were generally close to the foothills or near clear patches were food is accessible. Sites where individual bird data were collected were GPS marked and used only once to minimise, as much as possible, any pseudo replication.

We recorded videos (25fps, SONY® Handycam, HDR-XR250, SONY® Corporation, Japan) with the camera parallel and at a fixed height and position relative to the birds (*n* = 50) moving across level ground covered in snow at self-selected *U*. After filming, when the bird had moved out of shot, the camera was left recording, kept in its position and a 1 m scale bar was then held above each track way so it was visible on the video recording to determine actual speed for comparison with that calculated from the trackways. Bird speeds were calculated by analysing the videos using the Tracker® v. 5.0.5 (Open Source Physics) program to calculate speed as the distance moved for a given time. Only recordings where the bird was moving steadily were included in the study. To facilitate prediction of self-selected speed (*U*) using stride length (*l*_stride_), a photograph of each trackway was taken after filming from approximately 1.5 m directly above the tracks, individual strides were documented corresponding to the exact region of the track where the video recording was taken. Trackway photos were used for a direct comparison between speed from the video and trackway stride length (*l*_stride_). The mean of *l*_stride_ was measured from 1 to 5 strides using Image J v.1.50i. Data from the current study were also previously used to examine the differences between the kinematics of locomotion for freely moving ptarmigan in the field and from laboratory treadmill studies (see [1]).

### Data analyses

The relationship between leg kinematic parameters and *U* differs (i.e., the incremental change of *y* with *x*) depending on the gait used (e.g. [11, 13, 18, 75, 76]). Therefore, here, walking, grounded running and aerial running gaits were analysed separately. In a previous study [1] the relationship between *l*_stride_ and *U* was determined for free ranging male Svalbard ptarmigan. Here the same data were used, but this time *U* became the dependent variable and *l*_stride_ the independent variable to produce a gait specific predictive relationship (Fig. 1a). The sample sizes were *n* = 48 for walking, *n* = 56 for grounded running and *n* = 61 aerial running. Shapiro-Wilks tests were run on the residuals from the three regressions to ensure the data approximated a normal distribution, which they did in each case. To estimate speed using Alexander [10] and Thulborn and Wade [23] models, it is necessary to obtain hip height (*h*) for the ptarmigan. Unfortunately, we were not able to capture the birds, therefore we took the hip height estimate of 172.7 mm from literature [19]. To assess the accuracy of predictions of *U* derived from the three regression models for the 50 birds whose data were collected in this study, we estimated the percentage of error (Fig. 2) using the following:

4 $$ \%\mathrm{error}=\frac{\ \mathrm{predicted}\ u-\mathrm{measured}\ u}{\mathrm{measured}\ u} $$

Where predicted *U* is the estimate derived from the line of best fit based on the data from Marmol-Guijarro, Nudds [1] and measured *U* refers to the speed derived from the 50 new video recordings. All analyses were done in R v. 3.5.2 “Eggshell Igloo” [77].

## Data Availability

The datasets generated and/or analysed during the current study are available in the supplementary files associated with this manuscript.

## Abbreviations

*Fr*: Froude Number
*l*_stride_: Stride Length
*f*_stride_: Stride Frequency
*t*_stance_: Stance Phase
*t*_swing_: Swing Phase
*U*: Speed
*g*: Gravity
*h*: Functional Hip Height
SD: Standard Deviation

## References

[1] Marmol-Guijarro AC, Nudds RL, Marrin JC, Folkow LP, Codd JR (2019). Terrestrial locomotion of the Svalbard rock ptarmigan: comparing field and laboratory treadmill studies. Sci Rep.

[2] Pagano AM, Durner GM, Rode KD, Atwood TC, Atkinson SN, Peacock E, Costa DP, Owen MA, Williams TM (2018). High-energy, high-fat lifestyle challenges an Arctic apex predator, the polar bear. Science..

[3] Ratzlaff MH, Grant BD, Rathgeber-Lawrence R, Kunka KL (1995). Stride rates of horses trotting and cantering on a treadmill. J Equine Vet Sci.

[4] Williams TM, Ben-David M, Noren S, Rutishauser M, McDonald K, Heyward W (2002). Running energetics of the north American river otter: do short legs necessarily reduce efficiency on land?. Comp Biochem Physiol, Part A Mol Integr Physiol.

[5] Brockway JM, Gessaman JA (1977). The energy cost of locomotion on the level and on gradients for the red deer (*Cervus elaphus*). Q J Exp Physiol Cogn Med Sci.

[6] Bélanger M, Drew T, Provencher J, Rossignol S (1996). A comparison of treadmill locomotion in adult cats before and after spinal transection. J Neurophysiol.

[7] Herbin M, Hackert R, Gasc J-P, Renous S (2007). Gait parameters of treadmill versus overground locomotion in mouse. Behav Brain Res.

[8] Herbin M, Hommet E, Hanotin-Dossot V, Perret M, Hackert R (2018). Treadmill locomotion of the mouse lemur (*Microcebus murinus*); kinematic parameters during symmetrical and asymmetrical gaits. J Comp Physiol A.

[9] Mori S, Miyashita E, Nakajima K, Asanome M (1996). Quadrupedal locomotor movements in monkeys (*M. fuscata*) on a treadmill: kinematic analyses. Neuroreport..

[10] Alexander RM (1976). Estimates of speeds of dinosaurs. Nature..

[11] Gatesy SM, Biewener AA (1991). Bipedal locomotion: effects of speed, size and limb posture in birds and humans. J Zool.

[12] Abourachid A (2001). Kinematic parameters of terrestrial locomotion in cursorial (ratites), swimming (ducks), and striding birds (quail and Guinea fowl). Comp Biochem Physiol, Part A Mol Integr Physiol.

[13] Abourachid A, Renous S (2000). Bipedal locomotion in ratites (Paleognatiform) examples of cursorial birds. Ibis..

[14] Daley MA, Channon AJ, Nolan GS, Hall J (2016). Preferred gait and walk–run transition speeds in ostriches measured using GPS-IMU sensors. J Exp Biol.

[15] Nudds RL, Gardiner JD, Tickle PG, Codd JR (2010). Energetics and kinematics of walking in the barnacle goose (*Branta leucopsis*). Comp Biochem Physiol, Part A Mol Integr Physiol.

[16] Rose KA, Codd JR, Nudds RL (2016). Differential sex-specific walking kinematics in leghorn chickens (*Gallus Gallus domesticus*) selectively bred for different body size. J Exp Biol.

[17] Watson RR, Rubenson J, Coder L, Hoyt DF, Propert MWG, Marsh RL (2011). Gait-specific energetics contributes to economical walking and running in emus and ostriches. Proc R Soc B.

[18] Nudds RL, Folkow LP, Lees JJ, Tickle PG, Stokkan KA, Codd JR (2011). Evidence for energy savings from aerial running in the Svalbard rock ptarmigan (*Lagopus muta hyperborea*). Proc R Soc B.

[19] Lees JJ, Nudds RL, Folkow LP, Stokkan KA, Codd JR (2012). Understanding sex differences in the cost of terrestrial locomotion. Proc R Soc B.

[20] Lees JJ, Nudds R, Stokkan K-A, Folkow L, Codd J (2010). Reduced metabolic cost of locomotion in Svalbard rock ptarmigan (*Lagopus muta hyperborea*) during winter. PLoS One.

[21] Lees JJ, Stokkan K-A, Folkow LP, Codd JR (2012). Locomotor development in the Svalbard rock ptarmigan (*Lagopus muta hyperborea*). Polar Biol.

[22] Lee Y-N (1997). Bird and dinosaur footprints in the woodbine formation (Cenomanian). Texas Cretac Res.

[23] Thulborn RA, Wade M (1984). Dinosaur trackways in the Winton formation (mid-Creataceous) of Queensland. Mem Queensl Mus.

[24] McKeever PJ (1994). The behavioral and biostratigraphical significance and origin of vertebrate trackways from the Permian of Scotland. Palaios..

[25] Currie PJ, Eberth DA (2010). On gregarious behavior in *Albertosaurus*. Can J Earth Sci.

[26] Lockley MG, Nadon G, Currie PJ (2004). A diverse dinosaur-bird footprint assemblage from the lance formation, upper cretaceous, Eastern Wyoming: Implications for ichnotaxonomy. Ichnos.

[27] Warren JW, Wakefield NA (1972). Trackways of tetrapod vertebrates from the upper Devonian of Victoria, Australia. Nature.

[28] Clack JA (1997). Devonian tetrapod trackways and trackmakers; a review of the fossils and footprints. Palaeogeogr Palaeoclimatol Palaeoecol.

[29] Crompton RH, Pataky TC, Savage R, D'Août K, Bennett MR, Day MH, Bates K, Morse S, Sellers WI (2012). Human-like external function of the foot, and fully upright gait, confirmed in the 3.66 million year old Laetoli hominin footprints by topographic statistics, experimental footprint-formation and computer simulation. Interface Focus.

[30] Raichlen DA, Pontzer H, Sockol MD (2008). The Laetoli footprints and early hominin locomotor kinematics. J Hum Evol.

[31] Raichlen DA, Gordon AD, Harcourt-Smith WEH, Foster AD, Haas WR (2010). Laetoli footprints preserve earliest direct evidence of human-like bipedal biomechanics. PLoS One.

[32] McCrea RT, Buckley LG, Farlow JO, Lockley MG, Currie PJ, Matthews NA, Pemberton SG (2014). A ‘terror of tyrannosaurs’: the first trackways of Tyrannosaurids and evidence of gregariousness and pathology in Tyrannosauridae. PLoS One.

[33] González Riga BJ (2011). Speeds and stance of titanosaur sauropods: analysis of *Titanopodus* tracks from the late cretaceous of Mendoza, Argentina. An Acad Bras Cienc.

[34] Farlow JO (1981). Estimates of dinosaur speeds from a new trackway site in Texas. Nature..

[35] Day JJ, Norman DB, Upchurch P, Powell HP (2002). Dinosaur locomotion from a new trackway. Nature..

[36] Alexander RM, Jayes AS (1983). A dynamic similarity hypothesis for the gaits of quadrupedal mammals. J Zool.

[37] Thulborn RA (1984). Preferred gaits of bipedal dinosaurs. Alcheringa..

[38] Alexander RM, Langman VA, Jayes AS (1977). Fast locomotion of some African ungulates. J Zool.

[39] Hutchinson JR, Allen V (2009). The evolutionary continuum of limb function from early theropods to birds. Naturwissenschaften..

[40] Henderson D (2003). Footprints, trackways, and hip heights of bipedal dinosaurs—testing hip height predictions with computer models. Ichnos..

[41] Gatesy SM, Bäker M, Hutchinson JR (2009). Constraint-based exclusion of limb poses for reconstructing theropod dinosaur locomotion. J Vert Paleontol.

[42] Henderson DM (2006). Simulated weathering of dinosaur tracks and the implications for their characterization. Can J Earth Sci.

[43] Daley MA, Usherwood JR, Felix G, Biewener AA (2006). Running over rough terrain: Guinea fowl maintain dynamic stability despite a large unexpected change in substrate height. J Exp Biol.

[44] Daley MA, Biewener AA (2006). Running over rough terrain reveals limb control for intrinsic stability. Proc Natl Acad Sci U S A.

[45] Daley MA, Usherwood JR (2010). Two explanations for the compliant running paradox: reduced work of bouncing viscera and increased stability in uneven terrain. Biol Lett.

[46] Parker KL, Robbins CT, Hanley TA (1984). Energy expenditures for locomotion by mule deer and elk. J Wildl Manag.

[47] Fancy SG, White RG (1987). Energy expenditures for locomotion by barren-ground caribou. Can J Zool.

[48] Droghini A, Boutin S (2018). The calm during the storm: snowfall events decrease the movement rates of grey wolves (*Canis lupus*). PLoS One.

[49] Falkingham PL, Gatesy SM (2014). The birth of a dinosaur footprint: subsurface 3D motion reconstruction and discrete element simulation reveal track ontogeny. Proc Natl Acad Sci U S A.

[50] Sollas WJ (1879). On some three-toed footprints from the Triassic conglomerate of South Wales. Quart J Geol Soc London.

[51] Padian K, Olsen PE, Gillete DD, Lockley MG (1989). Ratite footprints and the stance and gait of Mesozoic theropords. Dinosaur tracks and traces.

[52] Gatesy SM, Middleton KM, Jenkins FA, Shubin NH (1999). Three-dimensional preservation of foot movements in Triassic theropod dinosaurs. Nature..

[53] Milàn J (2006). Variations in the morphology of emu (*Dromaius novaehollandiae*) tracks reflecting differences in walking pattern and substrate consistency: ichnotaxonomic implications. Palaeontology..

[54] Qiao M, Hughes M, Jindrich DL (2012). Response to medio-lateral perturbations of human walking and running.

[55] Qiao M, Jindrich DL (2014). Compensations during unsteady locomotion. Integr Comp Biol.

[56] Andrada E, Rode C, Blickhan R (2013). Grounded running in quails: simulations indicate benefits of observed fixed aperture angle between legs before touch-down. J Theor Biol.

[57] Cham R, Redfern MS (2002). Changes in gait when anticipating slippery floors. Gait Posture.

[58] Cappellini G, Ivanenko YP, Dominici N, Poppele RE, Lacquaniti F (2010). Motor patterns during walking on a slippery walkway. J Neurophysiol.

[59] Rainforth EC, Mazella M, Rainforth EC (2007). Estimating speeds of dinosaurs from trackways: a re-evaluation of assumptions. Contributions to the Paleontology of New Jersey (II)–Field and Guide Proceedings.

[60] Alexander RM (1991). Doubts and assumptions in dinosaur mechanics. Interdiscipl Sci Rev.

[61] Alexander RM (2006). Dinosaur biomechanics. Proc Biol Sci.

[62] Lees JJ, Gardiner J, Usherwood J, Nudds R (2016). Locomotor preferences in terrestrial vertebrates: an online crowdsourcing approach to data collection. Sci Rep.

[63] Paik IS, Huh M, Park KH, Hwang KG, Kim KS, Kim HJ (2006). Yeosu dinosaur track sites of Korea: the youngest dinosaur track records in Asia. J Asian Earth Sci.

[64] Abrahams M, Bordy EM, Sciscio L, Knoll F (2017). Scampering, trotting, walking tridactyl bipedal dinosaurs in southern Africa: ichnological account of a lower Jurassic palaeosurface (upper Elliot formation, Roma Valley) in Lesotho. Hist Biol.

[65] Carrano MT, Biewener AA (1999). Experimental alteration of limb posture in the chicken (*Gallus gallus*) and its bearing on the use of birds as analogs for dinosaur locomotion. J Morphol.

[66] Sellers WI, Manning PL (2007). Estimating dinosaur maximum running speeds using evolutionary robotics. Proc R Soc B.

[67] Day JJ, Norman DB, Gale AS, Upchurch P, Powell HP (2004). A middle Jurassic dinosaur trackway site from Oxfordshire, UK. Palaeontology.

[68] Rose KA, Tickle PG, Lees JJ, Stokkan K-A, Codd JR (2014). Neither season nor sex affects the cost of terrestrial locomotion in a circumpolar diving duck: the common eider (*Somateria mollissima*). Polar Biol.

[69] Rose KA, Nudds RL, Butler PJ, Codd JR (2015). Sex differences in gait utilization and energy metabolism during terrestrial locomotion in two varieties of chicken (*Gallus gallus domesticus*) selected for different body size. Biol Open.

[70] Rose KA, Bates KT, Nudds RL, Codd JR (2016). Ontogeny of sex differences in the energetics and kinematics of terrestrial locomotion in leghorn chickens (*Gallus gallus domesticus*). Sci Rep.

[71] Hutchinson JR (2004). Biomechanical modeling and sensitivity analysis of bipedal running ability. II Extinct taxa J Morphol.

[72] Nudds RL, Dyke GJ (2010). Narrow primary feather rachises in *Confuciusornis* and *Archaeopteryx* suggest poor flight ability. Science..

[73] Lees J, Garner T, Cooper G, Nudds R (2017). Rachis morphology cannot accurately predict the mechanical performance of primary feathers in extant (and therefore fossil) feathered flyers. R Soc Open Sci.

[74] Mortensen A, Unander S, Kolstad M, Blix AS (1983). Seasonal changes in body composition and crop content of Spitzbergen ptarmigan *Lagopus mutus hyperboreus*. Ornis Scand.

[75] Rubenson J, Heliams DB, Lloyd DG, Fournier PA (2004). Gait selection in the ostrich: mechanical and metabolic characteristics of walking and running with and without an aerial phase. Proc Biol Sci.

[76] Hancock JA, Stevens NJ, Biknevicius AR (2007). Whole-body mechanics and kinematics of terrestrial locomotion in the elegant-crested Tinamou *Eudromia elegans*. Ibis..

[77] R Core Team (2018). R: A Language and Environment for Statistical Computing. R version 3.5.2 ed.

